# Short-term effects of high-resolution (1-km) ambient PM_2.5_ and PM_10_ on hospital admission for pulmonary tuberculosis: a case-crossover study in Hainan, China

**DOI:** 10.3389/fpubh.2023.1252741

**Published:** 2023-09-05

**Authors:** Pan-Pan Zhu, Yi Gao, Gui-Zhong Zhou, Rui Liu, Xiao-Bo Li, Xian-Xian Fu, Jian Fu, Feng Lin, Yuan-Ping Zhou, Li Li

**Affiliations:** ^1^State Key Laboratory of Organ Failure Research, Department of Biostatistics, Guangdong Provincial Key Laboratory of Tropical Disease Research, School of Public Health, Southern Medical University, Guangzhou, Guangdong, China; ^2^Department of Infectious Disease and Hepatology Unit, Nanfang Hospital, Southern Medical University, Guangzhou, Guangdong, China; ^3^Department of Infectious Disease, Hainan General Hospital, Hainan Medical University, Haikou, Hainan, China; ^4^Department of Infectious Disease, The Second Affiliated Hospital, Hainan Medical University, Haikou, Hainan, China; ^5^Department of Neurosurgery, Haikou Municipal People’s Hospital and Central South University Xiangya Medical College Affiliated Hospital, Haikou, Hainan, China; ^6^Clinical Lab, Haikou Municipal People’s Hospital and Central South University Xiangya Medical College Affiliated Hospital, Haikou, Hainan, China; ^7^Department of Gastroenterology, Nanfang Hospital, Southern Medical University, Guangzhou, Guangdong, China

**Keywords:** particulate matter, hospitalization, pulmonary tuberculosis, case-crossover design, tropical region

## Abstract

**Introduction:**

There is limited evidence regarding particulate matter (PM)’s short-term effects on pulmonary tuberculosis (PTB) hospital admission. Our study aimed to determine the short-term associations of the exposure to ambient PM with aerodynamic diameters <2.5 μm (PM_2.5_) and < 10 μm (PM_10_) with hospital admission for PTB in Hainan, a tropical province in China.

**Methods:**

We collected individual data on patients hospitalized with PTB, PM_2.5_, PM_10_, and meteorological data from 2016 to 2019 in Hainan Province, China. Conditional logistic regression models with a time-stratified case-crossover design were used to assess the short-term effects of PM_2.5_ and PM_10_ on hospital admission for PTB at a spatial resolution of 1 km  ×  1 km. Stratified analyses were performed according to age at admission, sex, marital status, administrative division, and season of admission.

**Results:**

Each interquartile range (IQR) increases in the concentrations of PM_2.5_ and PM_10_ were associated with 1.155 (95% confidence interval [CI]: 1.041–1.282) and 1.142 (95% CI: 1.033–1.263) hospital admission risks for PTB at lag 0–8 days, respectively. The stratified analyses showed that the effects of PM_2.5_ and PM_10_ were statistically significant for patients aged ≥65 years, males, married, and those residing in prefecture-level cities. Regarding seasonal differences, the associations between PM and hospital admission for PTB were statistically significant in the warm season but not in the cold season. The effect of PM_2.5_ was consistently stronger than that of PM_10_ in most subgroups.

**Conclusion:**

Short-term exposure to PM increases the risk of hospital admission for PTB. The potential impact of PM with smaller aerodynamic diameter is more detrimental. Our findings highlight the importance of reducing ambient PM level to alleviate the burden of PTB.

## Introduction

Tuberculosis (TB) is a contagious infection caused by *Mycobacterium tuberculosis* (1). Transmission of TB occurs when an infected person passes microbial aerosols into the alveoli of a new host through coughing. Drainage from any site of active TB can be a source of infection (2). TB poses a significant threat to public health worldwide (3). In 2019, there were 10 million TB case notifications and TB was one of the top ten causes of death, resulting in 1.4 million deaths worldwide. The fourth Sustainable Development Goal is to end the TB epidemic by 2030. However, TB incidence declined by only 9% from 2015 to 2019 (i.e., from 142 to 130 per 100,000 individuals) (4). Global TB targets are off track with the incidence increasing from 127 to 134 per 100,000 individuals between 2020 and 2021 (5).

TB is a disease of poverty (6). There is an apparent socio-economic gradient in TB burden across different locations, with the poorest being at the highest risk (7). Common risk factors for TB include human immunodeficiency virus (HIV) infection, smoking, diabetes, alcohol abuse, undernutrition, and indoor air pollution (8). Outdoor air pollution may also affect TB infection and/or disease progression (9). Particulate matter (PM) with aerodynamic diameters <2.5 μm (PM_2.5_) and < 10 μm (PM_10_) are the two main ambient air pollutants. PM_10_ can be deposited in the bronchi and PM_2.5_ can further reach the alveoli. The adverse impacts of the PM exposure on human health have been comprehensively discussed in epidemiological, experimental, and mechanism studies (10–13). As for the impact of outdoor exposure to PM on TB, a few studies have suggested that short-term exposure to ambient PM_2.5_ is positively associated with TB incidence (14–16), while others have reported a statistically non-significant association (17, 18). Similarly, the short-term association between exposure to PM_10_ and TB incidence has also been inconclusive (19–21). TB is a chronic infectious disease. Patients with relatively severe TB symptoms must be hospitalized. However, previous studies have seldom explored the association between PM exposure and hospital admission for TB (22).

China has a high TB burden, ranking third following India and Indonesia in 2019. In that year, 833,000 individuals (i.e., 58.3 per 100,000 individuals) developed TB in China, accounting for 8.4% of the global population (4). Of these, 775,764 had pulmonary tuberculosis (PTB), representing 93.1% of the total number of patients with TB in China (23). Hainan Province is a relatively less developed province in China. From 2016 to 2019, the PTB case notification rate in Hainan Province increased from 76.5 to 90.3 per 100,000 individuals, with an average annual increase of >4.0% (24). Further, Hainan Province had China’s fifth-highest notification rate of PTB cases in 2019. Probing the association between PM exposure and hospital admission for PTB in Hainan Province would enrich our knowledge of the impact of PM on population health in tropical areas, shed light on targeted intervention measures, and help reduce the PTB burden more effectively. Herein, we aimed to determine the associations of hospital admission for PTB with PM_2.5_ and PM_10_, and further identify the potentially vulnerable populations in Hainan Province, China from 2016 to 2019.

## Methods

### Study area

Hainan Province lies in the south of China between latitude 3°35′ to 20°10’N and longitude 108°37′ to 118°45′E with a land area of 35,354 square kilometers. In 2019, the total resident population of Hainan Province was 9,447,200 (25). From 2016 to 2019, PM_2.5_ and PM_10_ exceeded the levels of 75 μg/m^3^ and 150 μg/m^3^ (class two standard according to Chinese National Ambient Air Quality Standards [GB3095-2012]) in 0.6 and 0% of the days, respectively, which was lower than the national average (12.5, 7.7%).

### Data collection

We obtained the individual data of all PTB patients hospitalized in the top three largest hospitals that serve PTB patients in Hainan Province (i.e., Hainan General Hospital, The Second Affiliated Hospital of Hainan Medical University, and Haikou Municipal People’s Hospital) from January 1st, 2016 to December 31st, 2019. The collected data included the date of admission, age, sex, marital status, residential address, and primary diagnosis, coded according to the International Statistical Classification of Diseases and Related Health Problems, 10th edition (ICD-10). We included all patients whose primary discharge diagnosis was PTB (ICD-10 codes A15–A16). This study was approved by the Research Ethics Committee of Hainan General Hospital (No. 2021–314).

Data on daily average concentrations of PM_2.5_ and PM_10_ with a spatial resolution of 1 km × 1 km were obtained from the National Earth System Science Data Center, National Science & Technology Infrastructure of China.^1^ Daily meteorological data (i.e., mean temperature and relative humidity) were downloaded from the National Meteorological Information Center.^2^

### Study design and exposure assignment

A time-stratified case-crossover design was applied in this study to assess the short-term effects of PM_2.5_ and PM_10_ on hospital admission for PTB. For each hospital admission, the case day was the day of admission, whereas the control day was the same day of other weeks within the same month. This self-pairing case–control design has the unique advantage of controlling for long-term and seasonal trends as well as time-invariant subject-specific variables or those that change slowly over time.

**Figure 1:**
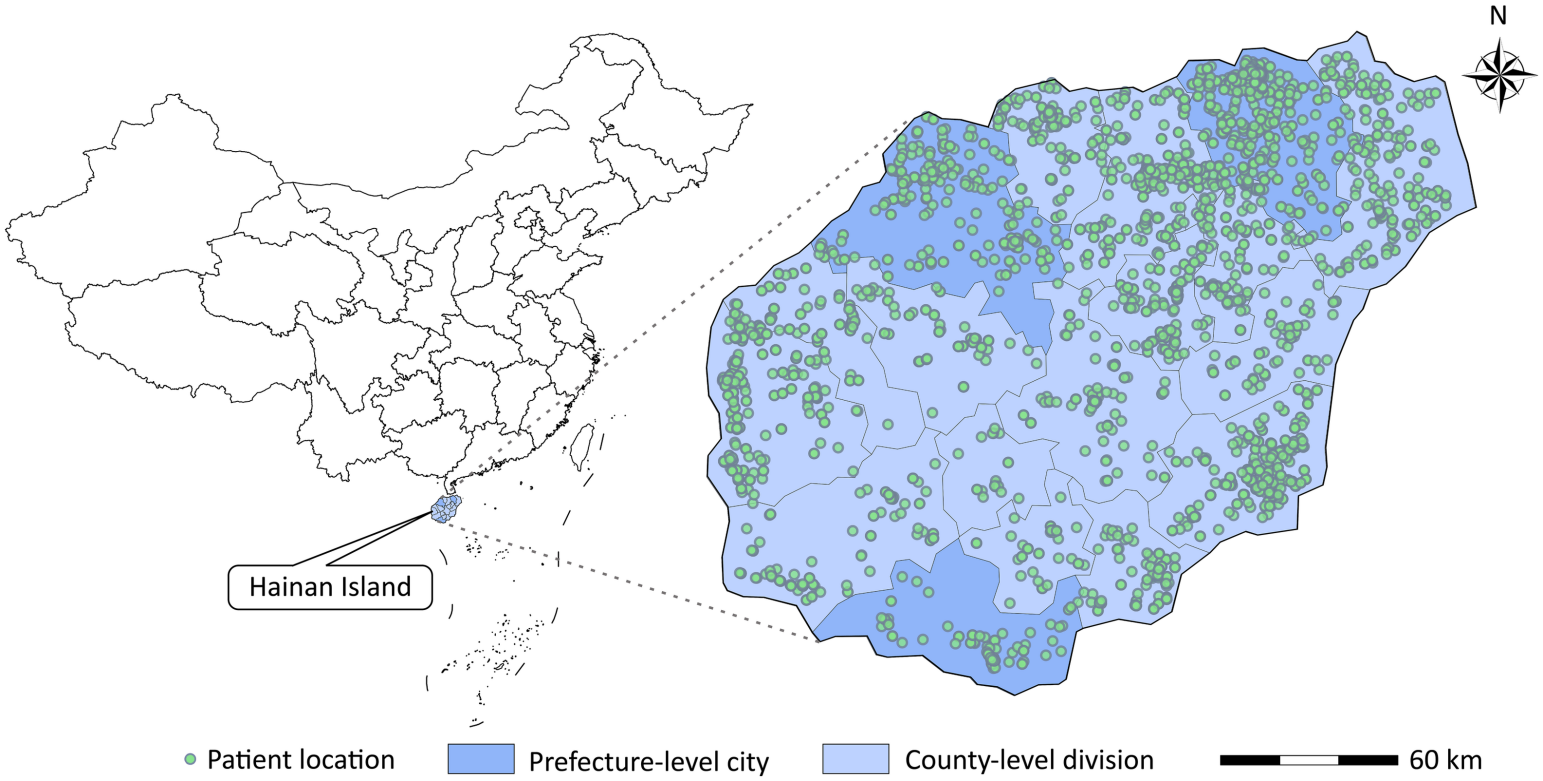
Locations of patients admitted to the top three largest hospitals in Hainan for pulmonary tuberculosis.

In addition, we matched each patient to the nearest 1 km × 1 km PM grid and meteorological monitoring station based on the patient’s residential address (Figure 1), generating 2,157 strata of exposure proxies for PM and meteorological factors (including mean temperature and relative humidity). Patients shared the same exposure proxy if they were in the same stratum.

### Statistical analysis

Conditional logistic regression models were used to investigate the effects of PM_2.5_ and PM_10_ on hospital admission for PTB. The model is expressed as follows:


$$\begin{array}{c} \log it\;\left(P_{i}\right)=\alpha_{i}+\beta PM+NS\left(Temp_{0-14},df=3\right)\\ +NS\left(RH,df=3\right)+\delta Holiday \end{array}$$

Where $P_{i}$ is the probability of hospital admission for PTB in stratum *i*. $\alpha_{i}$ is the intercept for stratum i. Here, we considered single-day lags from 0 to 14 days and multi-day lags from 0–1 to 0–14 days (0–1 and 0–14 days mean the 2–day and 15–day moving average, respectively) for the concentration of PM (i.e., PM_2.5_ or PM_10_) (26). We added each lag term to the model separately and selected the lags corresponding to the models with the strongest effects of PM_2.5_ and PM_10_ (27–29). Many previous studies have suggested that the effect of temperature on health outcomes is limited to 2 weeks (30–32). In addition, previous studies controlled for the potential confounding effects of current–day relative humidity when assessing the health effects of air pollution (28, 33). Therefore, natural cubic splines with three degrees of freedom (*df*s) were applied to the 15–day moving average temperature and current-day relative humidity. An indicator variable for holidays is also included in the model. The reported effects for the risk of PTB hospitalization were expressed as odds ratios (ORs) and corresponding 95% confidence intervals (CIs), which were associated with each interquartile range (IQR) increase in PM concentration.

We classified Hainan Province into two administrative regions: prefecture-level cities and county-level divisions (including county-level cities, counties, and autonomous counties). Stratified analyses were conducted by age at admission (<65 and ≥ 65 years), sex, marital status (married and others), administrative division (prefecture-level city and county-level division), and season of admission (warm [May–October] and cold [November–April]). Differences in the estimated effects of PM between subgroups were tested using a *Z* test (34):


$$Z=\frac{\beta_{1}-\beta_{2}}{\sqrt{SE^{2}\left(\beta_{1}\right)+SE^{2}\left(\beta_{2}\right)}}$$

Where *β*_1_ and *β*_2_ are the regression coefficients of PM for two comparative subgroups. *SE* (*β*_1_) and *SE* (*β*_2_) are the standard errors of *β*_1_ and *β*_2_, respectively.

Sensitivity analyses were performed to verify the robustness of the findings by changing the *df*s of the natural cubic splines for temperature and relative humidity to 4–6. Two-sided *p* < 0.05 was regarded as statistically significant. R software version 4.2.2 (R Foundation for Statistical Computing) was used for all analyses.

## Results

Table 1 shows the number and percentage of hospital admission for PTB in different subgroups in Hainan Province from 2016 to 2019. A total of 4,562 hospital admission for PTB were included in this study, all of which occurred on Hainan Island (Figure 1). Patients aged < 65, male, married, and those living in county-level divisions accounted for 76.2, 76.6, 79.7, and 67.7% of admissions, respectively. There were slightly more patients in the warm season (54.0%) than in the cold season. The average concentrations of PM_2.5_ and PM_10_ were 20.2 μg/m^3^ and 36.2 μg/m^3^, respectively during the study period (Table 2).

**Table 1:** Number and percentage of PTB hospitalization in different subgroups in Hainan Province, China, 2016–2019.

Subgroups	*N* (%)
**Age of admission, years**	
<65	3,476 (76.2)
≥65	1,086 (23.8)
**Sex**	
Male	3,496 (76.6)
Female	1,066 (23.4)
**Marital status**	
Married	3,637 (79.7)
Others	925 (20.3)
**Administrative division**	
Prefecture-level city	1,473 (32.3)
County-level division	3,089 (67.7)
**Season of admission**	
Warm	2,462 (54.0)
Cold	2,100 (46.0)

PTB, pulmonary tuberculosis.

**Table 2:** Summary statistics of PM_2.5_, PM_10_, and meteorological factors in Hainan Province, China, 2016–2019.

Variables	Mean	Minimum	P25	Median	P75	Maximum	SD
PM_2.5_ (μg/m^3^)	20.2	1.6	12.6	18.4	25.5	122.8	9.7
PM_10_ (μg/m^3^)	36.2	7.7	26.3	34.0	43.2	145.5	13.0
Mean temperature (°C)	24.9	6.0	22.2	25.9	28.2	33.0	4.4
Relative humidity (%)	81.9	37.0	77.0	82.0	88.0	100.0	8.1

PM_2.5_, particulate matter with aerodynamic diameters < 2.5 μm; PM_10_, particulate matter with aerodynamic diameters < 10 μm; SD, standard deviation; P25, the 25th percentile; P75, the 75th percentile.

**Figure 2:**
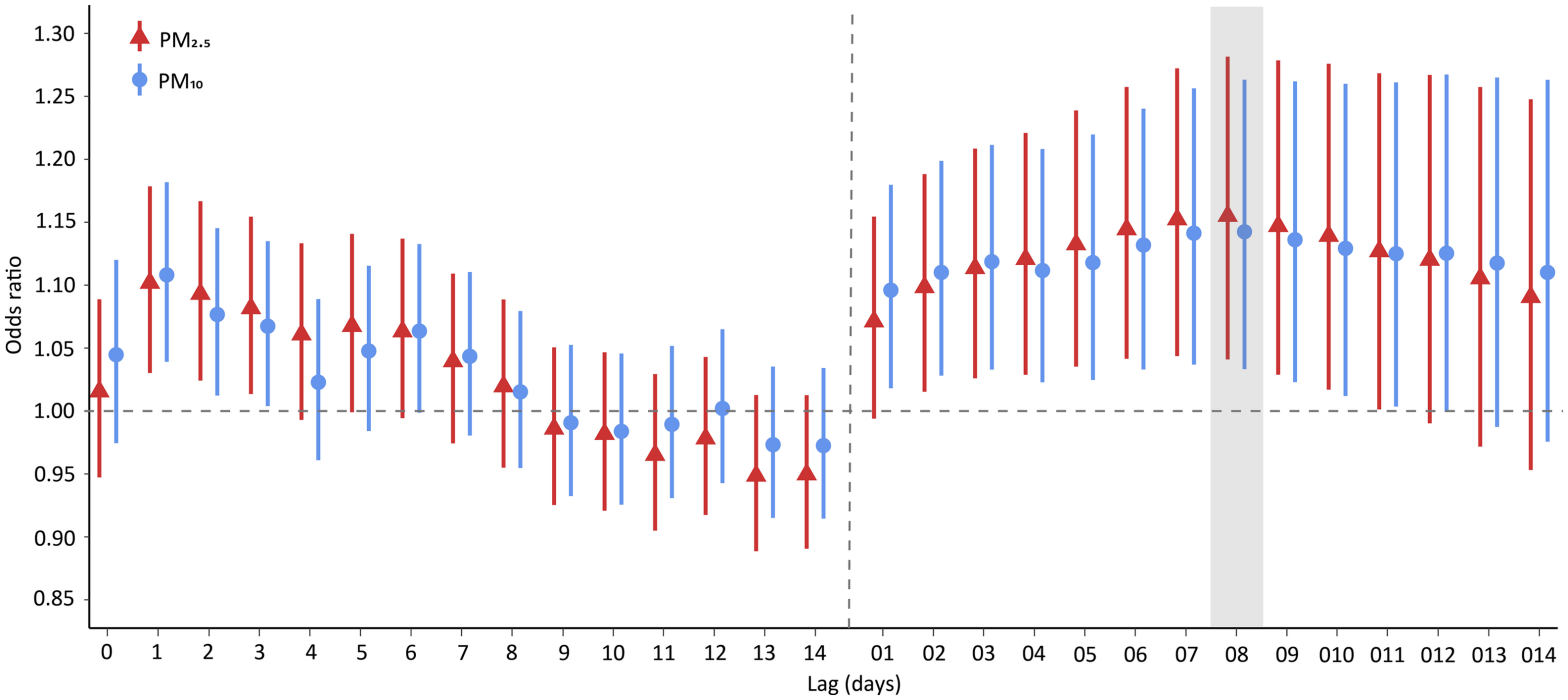
Odds ratios of PTB hospitalization associated with an interquartile range increase in PM_2.5_ and PM_10_ concentration. Points are the point estimates of odds ratios, while lines represent the corresponding 95% confidence intervals. Lag 01-014 are moving averages of the concentrations of PM_2.5_ and PM_10_ over 0–1 to 0–14 days. PTB, pulmonary tuberculosis; PM_2.5_, particulate matter with aerodynamic diameters <2.5 μm; PM_10_, particulate matter with aerodynamic diameters <10 μm.

Figure 2 illustrates the *OR*s of hospital admission for PTB associated with each IQR increase in PM_2.5_ and PM_10_ across various lag days. We found that the effect of PM_2.5_ at lag 0–8 days was the highest among the effects at different lags, and the same finding was observed for PM_10._ Therefore, we mainly explored the effects of PM_2.5_ and PM_10_ at a lag of 0–8 days. Specifically, the risk of PTB hospitalization were, respectively, 1.155 (95% confidence interval [CI]: 1.041–1.282) and 1.142 (95% CI: 1.033–1.263) for an IQR increase in PM_2.5_ and PM_10_ at a lag of 0–8 days.

**Figure 3:**
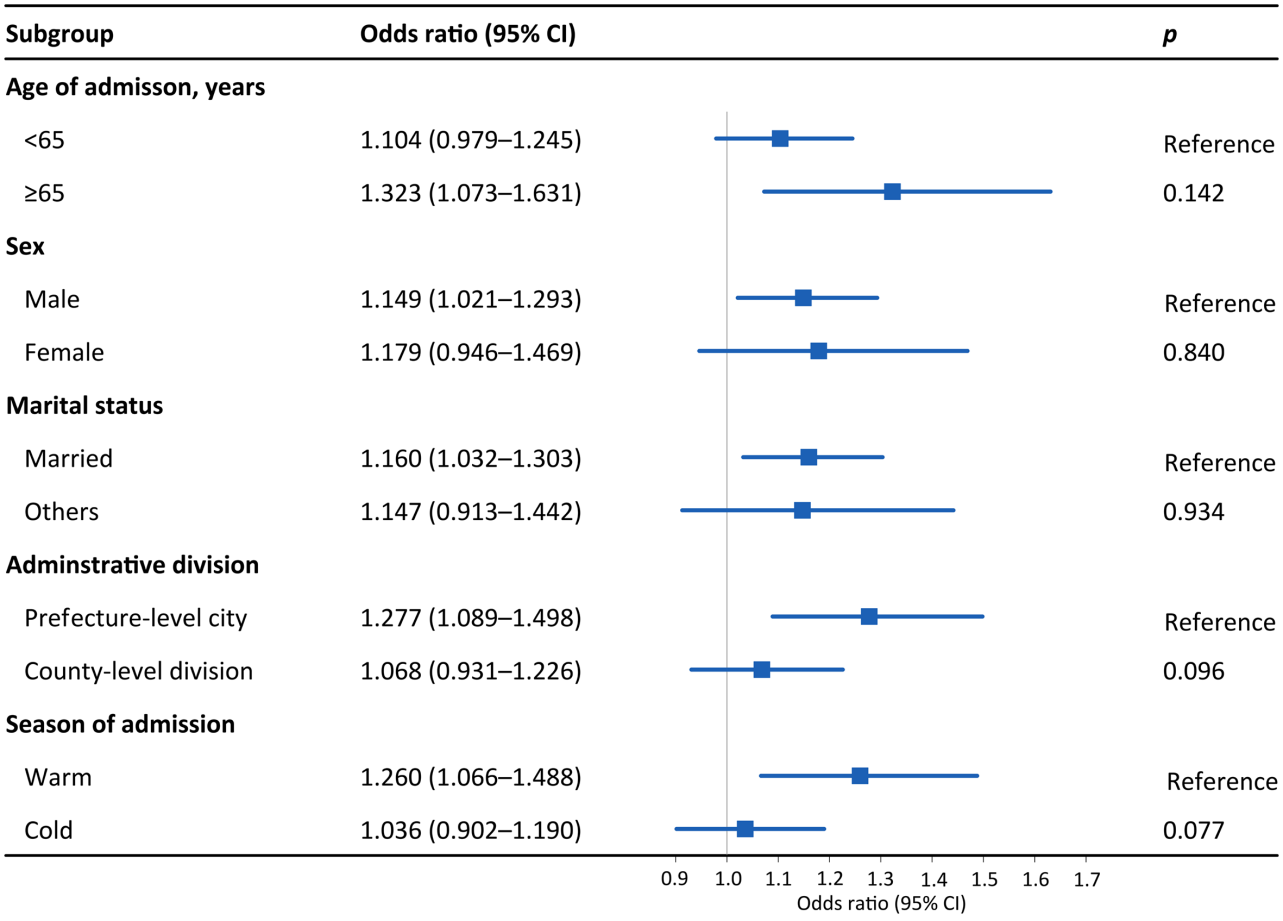
Odds ratios for PTB hospitalization among subgroups associated with an interquartile range increase in PM_2.5_ concentration. Subgroups were stratified by age, sex, marital status, administrative division, and season. *p* values were obtained with *Z* tests. Points are the point estimates of odds ratios, while lines represent the corresponding 95% confidence intervals. PTB, pulmonary tuberculosis; PM_2.5_, particulate matter with aerodynamic diameters <2.5 μm; CI, confidence interval.

**Figure 4:**
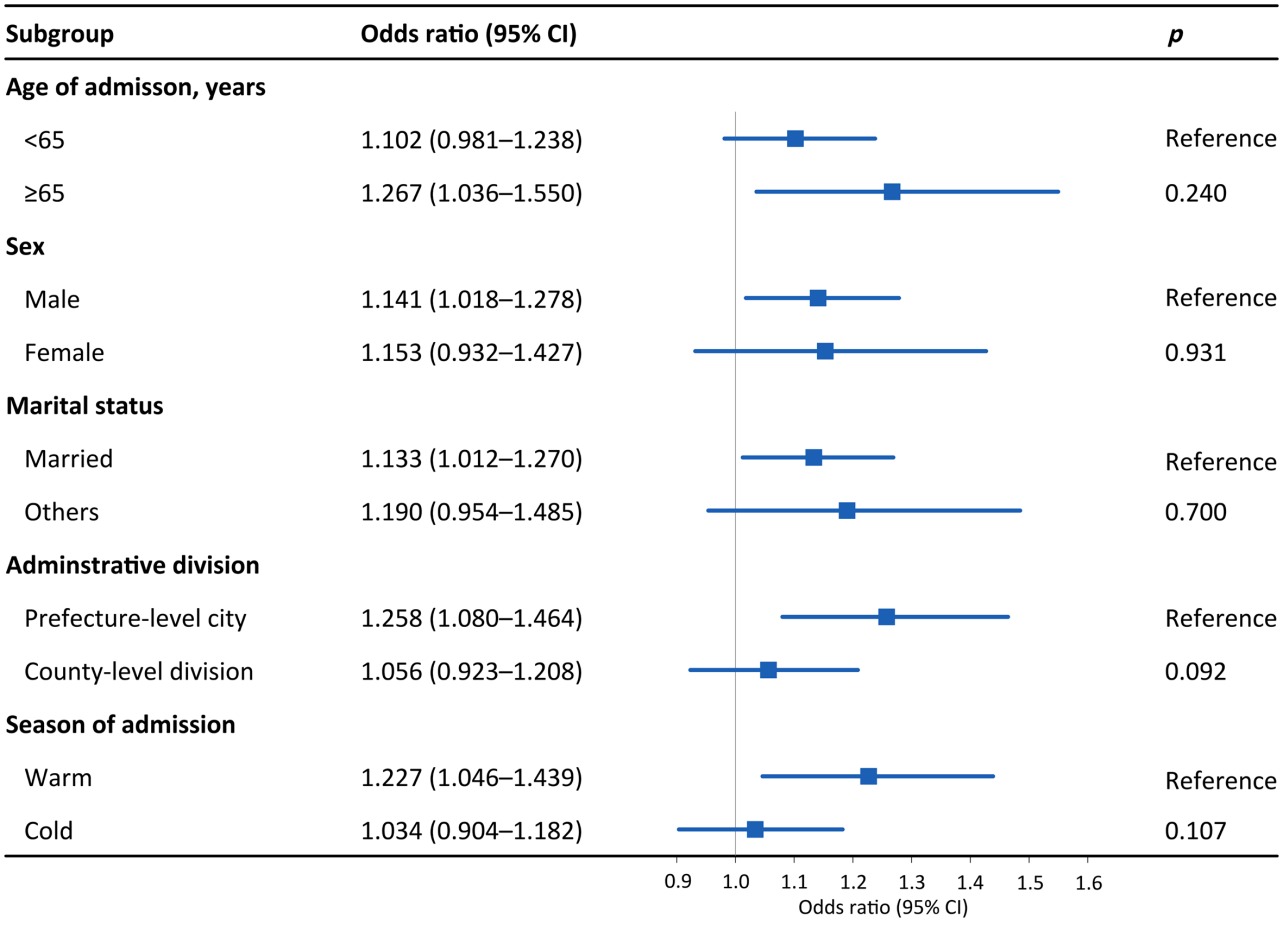
Odds ratios for PTB hospitalization among subgroups associated with an interquartile range increase in PM_10_ concentration. Subgroups were stratified by age, sex, marital status, administrative division, and season. *p* values were obtained with *Z* tests. Points are the point estimates of odds ratios, while lines represent the corresponding 95% confidence intervals. PTB, pulmonary tuberculosis; PM_10_, particulate matter with aerodynamic diameters <10 μm; CI, confidence interval.

Figure 3 presents the effects of PM_2.5_ on hospital admission for PTB in different subgroups. An IQR increase in PM_2.5_ was associated with 1.323 (95% CI: 1.073–1.631), 1.149 (95% CI: 1.021–1.293), 1.160 (95% CI: 1.032–1.303), and 1.277 (95% CI: 1.089–1.498) hospital admission risks for PTB among patients aged ≥65 years, male patients, married individuals, and those living in prefecture-level cities, respectively. As for the disparity by season, the effect of PM_2.5_ on hospital admission for PTB was statistically significant in the warm season (OR = 1.260, 95% CI: 1.066–1.488) but not in the cold season. The trends in the differences in the effects of PM_10_ across subgroups were similar to those of PM_2.5_, although the subgroup differences were not statistically significant (*p* > 0.05; Figures 3, 4). The effects of PM_2.5_ on hospital admission for PTB seemed to be greater than those of PM_10_ for most of the subgroups.

The sensitivity analysis results suggested that the main analysis findings were robust when changing the *df*s for the natural cubic splines of temperature and relative humidity to 4–6 (Supplementary Tables S1, S2).

## Discussion

The present study evaluated the short-term effects of ambient PM_2.5_ and PM_10_ exposure on hospital admission for PTB in a tropical Chinese province. It was found that both PM_2.5_ (OR = 1.155) and PM_10_ (OR = 1.142) were positively associated with hospital admission for PTB, which was partially in line with the findings of previous studies that reported adverse effects of PM on PTB outcomes, such as incidence (19, 35) and mortality (36). However, Álvaro-Meca et al. found that the effect of PM_10_ on hospital admission for PTB was not statistically significant in HIV-infected patients (22). The health impact of PM on PTB hospitalization may vary across different populations, warranting further investigation.

The mechanism linking PM exposure to hospital admission for PTB remains unclear, however, several plausible explanations exist. Fine PM can irritate the lung tissue, damage the tracheobronchial mucosa, and impair the body’s anti-mycobacterial immunity by inhibiting the synthesis and secretion of various key inflammatory mediators in the fight against *M. tuberculosis* (37). The susceptibility and infection of *M. tuberculosis* may be aggravated when exposed to high concentrations of PM because it induces alterations in protective host immune responses (38). Meanwhile, an experimental study suggested that exposure to PM_2.5_ and PM_10_ increased the intracellular growth of *M. tuberculosis* (39). Therefore, PM exposure may exacerbate *M. tuberculosis* infections in the lungs and increase risk of hospital admission for PTB.

We found that the effect of PM_2.5_ on hospital admission for PTB was higher than that of PM_10_, which was consistent with previous studies on respiratory disease hospitalizations (28, 40). An experimental study also reported that PM_2.5_ induced higher oxidative stress and more malondialdehyde in rat lung epithelial cells and cells treated with PM_2.5_ were more susceptible to apoptosis than PM_10_ (41). The stronger effect of PM_2.5_ on PTB outcomes may be attributed to its physicochemical characteristics such as a larger surface area and longer atmospheric suspension (42). At the same time, PM_2.5_ can carry external impurities, such as metal ions, minerals, and polycyclic aromatic hydrocarbons into the fine bronchi and alveoli (43, 44), which may lead to various forms of inflammatory responses and impair cellular immune function in the lungs (45, 46).

In accordance with previous research (16), we found that patients aged ≥65 years were vulnerable to PM exposure. Older adults infected with *M. tuberculosis*, a potent stimulator of numerous inflammatory cytokines, tend to develop a range of inflammatory responses (47). With a decline in the innate immune response, PM inhalation may lead to overwhelming inflammation when superimposed on the inflammation associated with aging in the older adults patients with PTB (48).

Consistent with a previous study on TB incidence conducted in Shanghai (16), we found that PM significantly affected hospital admission for PTB in male patients but not in female patients. Polymorphisms of the Toll-like receptor 8 gene (gene identity: 51311) on the X chromosome are associated with susceptibility to PTB in males, and susceptibility to PTB also increases in male carriers of rs3764880 allele A (49). Additionally, males tend to consume alcohol and nicotine (50), which may have a significant inhibitory effect on tumor necrosis factor-α production and cell-mediated immunity, thereby exacerbating the PTB progression (51).

Our study found that the association between PM and hospital admission for PTB was statistically significant for married patients but not for others. Married people have less time to perform stress-relieving healthy physical activity for family-related tasks, including childcare (52). An Indian study found that single TB patients had higher quality of life scores (53). A cross-sectional study of treated PTB patients suggested that married PTB patients had lower mental-domain scores (54). Less physical activity, lower quality of life, and poor mental health may weaken the immune system, in which case PM inhalation may have more harmful health effects.

In our study, the effect of PM on hospital admission for PTB varied according to administrative division, with PM having a greater impact on patients living in prefecture-level cities. This finding is similar to that of a national study in China that explored the associations between major PM_2.5_ components and TB incidence and mortality (55). Inconsistent socioeconomic characteristics across administrative regions may explain this result. The higher population density of prefecture-level cities (Supplementary Table S3) may facilitate the spread of respiratory viruses, such as influenza (56). Higher respiratory virus incidence and ambient PM concentration may exacerbate PTB (20, 38). Therefore, the effect of PM in prefecture-level cities tends to be more detrimental than that in county-level divisions.

We found that the effect of PM on hospital admission for PTB was statistically significant in the warm season but not in the cold season. The result can be interpreted as follows: First, *M. tuberculosis*’s rate of proliferation and growth increases significantly with increasing temperature (57). Second, on average, people spend more time outdoors (or with windows open) during the warm season, which can expose them to outdoor PM more often. Third, PM may reduce the phagocytosis of *M. tuberculosis* by peripheral blood mononuclear cells in warmer months but not in colder months (58).

### Limitations

Our study has a few limitations. First, we included 4,562 PTB hospital admissions. Although the participating hospitals are the top three largest hospitals and the PTB patients came from 3/4 prefecture-level cities and all 15 county-level divisions directly under the provincial government in Hainan, the sample size may be insufficient for the statistical inference of the association between exposure to ambient PM and hospital admission for PTB in some subgroups. More reliable results can be obtained using data from more hospital patients. Second, we only examined the association between PTB hospital admission with PM_2.5_ and PM_10_. It will be interesting to further assess the effects of PM_2.5_ components and PM_1_. Third, measurement errors may exist in the exposure proxy for the PM concentration. Nevertheless, we used PM data with 1 km × 1 km resolution in this study. Effect estimates derived from such exposure data may be more accurate than those based on data collected from only a few monitoring stations. Finally, this ecological study sheds light on the link between PM exposure and hospital admission for PTB, however, the underlying mechanisms of this link warrant further investigation.

## Conclusion

Taken together, exposure to ambient PM_2.5_ and PM_10_ increases the risk of PTB hospital admission. PM with a smaller aerodynamic diameter is more hazardous. Our findings highlight the importance of reducing ambient PM concentrations to alleviate the PTB burden.

## Data availability statement

The data sets generated during and/or analyzed during the current study are available from the corresponding authors on reasonable request.

## Ethics statement

This study was approved by the Research Ethics Committee of Hainan General Hospital (No. 2021-314). Written informed consent was not required as the data was anonymized for the study. Consent was waived by the Research Ethics Committee.

## Author contributions

P-PZ: formal analysis, methodology, software, visualization, and writing – original draft. YG: conceptualization, funding acquisition, investigation, project administration, resources, validation, and writing – original draft. G-ZZ: resources and writing – original draft. RL, X-BL, X-XF, JF, and FL: data curation. Y-PZ: conceptualization, supervision, and writing – review and editing. LL: conceptualization, funding acquisition, methodology, supervision, and writing – review and editing. All authors contributed to the article and approved the submitted version.

## Funding

This work was supported by the National Natural Science Foundation of China [82003555] and Hainan Province Science and Technology Special Fund [ZDYF2021SHFZ079].

## Conflict of interest

The authors declare that the research was conducted in the absence of any commercial or financial relationships that could be construed as a potential conflict of interest.

## Publisher’s note

All claims expressed in this article are solely those of the authors and do not necessarily represent those of their affiliated organizations, or those of the publisher, the editors and the reviewers. Any product that may be evaluated in this article, or claim that may be made by its manufacturer, is not guaranteed or endorsed by the publisher.

## Footnotes

^1^ http://www.geodata.cn

^2^ http://data.cma.cn/en
