# Supplementary material for: Short-term effects of high-resolution (1-km) ambient PM2.5 and PM10 on hospital admission for pulmonary tuberculosis: a case-crossover study in Hainan, China
Source: Front Public Health. 2023 Sep 5;11:1252741. doi: 10.3389/fpubh.2023.1252741 (PMC10509552; doi:10.3389/fpubh.2023.1252741)
Supplement: Supplementary file 1 [file Data_Sheet_1.docx]

**Supplemental Materials**

**Short-term effects of high-resolution (1-km) ambient PM_2.5_ and PM_10_ on** **hospital admission for pulmonary tuberculosis:** **a case-crossover study in Hainan, China**

Pan-Pan Zhu^1#^, Yi Gao^2,3#^, Gui-Zhong Zhou^4#,^ Rui Liu^4^, Xiao-Bo Li^5^, Xian-Xian Fu^6^, Jian Fu^3^, Feng Lin^3^, Yuan-Ping Zhou^2,7*^, Li Li^1*^

^1^State Key Laboratory of Organ Failure Research, Department of Biostatistics, Guangdong Provincial Key Laboratory of Tropical Disease Research, School of Public Health, Southern Medical University, Guangzhou, 510515, Guangdong, China

^2^Department of Infectious Disease and Hepatology unit, Nanfang Hospital, Southern Medical University, Guangzhou, 510515, Guangdong, China

^3^Department of Infectious Disease, Hainan General Hospital, Hainan Medical University, Haikou,570311, Hainan, China

^4^Department of Infectious Disease, The Second Affiliated Hospital, Hainan Medical University, Haikou, 570105, Hainan, China

^5^Department of Neurosurgery, Haikou Municipal People's Hospital and Central South University Xiangya Medical College Affiliated Hospital, Haikou, 570208, Hainan, China,

^6^Clinical Lab, Haikou Municipal People's Hospital and Central South University Xiangya Medical College Affiliated Hospital, Haikou, China, Haikou, 570208, Hainan, China

^7^Department of Gastroenterology, Nanfang Hospital, Southern Medical University, Guangzhou, 510515, Guangdong, China

^#^ Contributed equally as co-first authors

* Contributed equally as co-corresponding authors:

Li Li (Email: lylygdsg@163.com)

Yuan-Ping Zhou (Email: yuanpingzhou@163.com)

**OUTLINE**

| **Title** | **Page** |
| --- | --- |
| **Table S1.** Association between PTB hospitalization and PM_2.5_ when changing *df*s for temperature and humidity from three to six. | **1** |
| **Table S2.** Association between PTB hospitalization and PM_10_ when changing *df*s for temperature and humidity from three to six | **2** |
| **Table S3.** Population densities in different administrative regions of Hainan Province, China, 2016–2019. | **3** |

**Table S1.** Associations between PTB hospitalization and PM_2.5_ when changing degrees of freedom for temperature and humidity from three to six.

| Degrees of freedom | Odds ratio (95% confidence interval)^a^ |
| --- | --- |
| 3^b^ | 1.155 (1.041–1.282) |
| 4 | 1.156 (1.042–1.283) |
| 5 | 1.156 (1.041–1.282) |
| 6 | 1.159 (1.044–1.286) |

^a^ Odds ratios of PTB hospitalization associated with per interquartile range (IQR) increase in PM_2.5_ at lag 0–8 days.

^b^ Three degrees of freedom were applied in the main analysis.

Abbreviations: PTB, pulmonary tuberculosis; PM_2.5_$,$ particulate matter with aerodynamic diameters <2.5μm.

**Table S2.** Associations between PTB hospitalization and PM_10_ when changing degrees of freedom for temperature and humidity from three to six.

| Degrees of freedom | Odds ratio (95% confidence interval)^a^ |
| --- | --- |
| 3^b^ | 1.142 (1.033–1.263) |
| 4 | 1.143 (1.034–1.264) |
| 5 | 1.143 (1.033–1.264) |
| 6 | 1.147 (1.037–1.269) |

^a^ Odds ratios of PTB hospitalization associated with per interquartile range (IQR) increase in PM_10_ at lag 0–8 days.

^b^ Three degrees of freedom were applied in the main analysis.

Abbreviations: PTB, pulmonary tuberculosis; PM_10_$,$ particulate matter with aerodynamic diameters <10μm.

**Table S3.** Population densities in different administrative regions of Hainan Province, China, 2016–2019.

| Administrative region | Population density (per km^2^) | | | | Annual average  (per km^2^) |
| --- | --- | --- | --- | --- | --- |
|  | 2016 | 2017 | 2018 | 2019 |  |
| Prefecture-level city | 531.2 | 537.5 | 543.5 | 549.5 | 540.4 |
| County-level division | 202.1 | 203.7 | 205.3 | 207.6 | 204.7 |
